# A Novel Translational Ovine Pulmonary Adenocarcinoma Model for Human Lung Cancer

**DOI:** 10.3389/fonc.2019.00534

**Published:** 2019-06-19

**Authors:** Mark E. Gray, Paul Sullivan, Jamie R. K. Marland, Stephen N. Greenhalgh, James Meehan, Rachael Gregson, R. Eddie Clutton, Chris Cousens, David J. Griffiths, Alan Murray, David Argyle

**Affiliations:** ^1^The Royal (Dick) School of Veterinary Studies and Roslin Institute, University of Edinburgh, Easter Bush, Edinburgh, United Kingdom; ^2^Cancer Research UK Edinburgh Centre and Division of Pathology Laboratories, Institute of Genetics and Molecular Medicine, University of Edinburgh, Edinburgh, United Kingdom; ^3^School of Engineering, Institute for Integrated Micro and Nano Systems, Edinburgh, United Kingdom; ^4^Institute of Sensors, Signals and Systems, School of Engineering and Physical Sciences, Heriot-Watt University, Edinburgh, United Kingdom; ^5^Moredun Research Institute, Pentlands Science Park, Midlothian, United Kingdom

**Keywords:** human lung cancer, ovine pulmonary adenocarcinoma, novel translational lung cancer model, pre-clinical research, computed tomography-guided sensor implantation

## Abstract

*In vitro* cell line and *in vivo* murine models have historically dominated pre-clinical cancer research. These models can be expensive and time consuming and lead to only a small percentage of anti-cancer drugs gaining a license for human use. Large animal models that reflect human disease have high translational value; these can be used to overcome current pre-clinical research limitations through the integration of drug development techniques with surgical procedures and anesthetic protocols, along with emerging fields such as implantable medical devices. Ovine pulmonary adenocarcinoma (OPA) is a naturally-occurring lung cancer that is caused by the jaagsiekte sheep retrovirus. The disease has similar histological classification and oncogenic pathway activation to that of human lung adenocarcinomas making it a valuable model for studying human lung cancer. Developing OPA models to include techniques used in the treatment of human lung cancer would enhance its translational potential, making it an excellent research tool in assessing cancer therapeutics. In this study we developed a novel OPA model to validate the ability of miniaturized implantable O_2_ and pH sensors to monitor the tumor microenvironment. Naturally-occurring pre-clinical OPA cases were obtained through an on-farm ultrasound screening programme. Sensors were implanted into OPA tumors of anesthetized sheep using a CT-guided trans-thoracic percutaneous implantation procedure. This study reports the findings from 9 sheep that received sensor implantations. Time taken from initial CT scans to the placement of a single sensor into an OPA tumor was 45 ± 5 min, with all implantations resulting in the successful delivery of sensors into tumors. Immediate post-implantation mild pneumothoraces occurred in 4 sheep, which was successfully managed in all cases. This is, to the best of our knowledge, the first description of the use of naturally-occurring OPA cases as a pre-clinical surgical model. Through the integration of techniques used in the treatment of human lung cancer patients, including ultrasound, general anesthesia, CT and surgery into the OPA model, we have demonstrated its translational potential. Although our research was tailored specifically for the implantation of sensors into lung tumors, we believe the model could also be developed for other pre-clinical applications.

## Introduction

The process of developing and validating new anti-cancer agents typically follows a step-wise process from *in vitro* and *in vivo* testing through to phase I, II, and III clinical trials. It has been estimated that several hundred million dollars and up to 10 years of research is needed to take a drug from its initial concept to the completion of phase III trials (1). Pharmaceutical companies may be discouraged from developing new cancer drugs, not only due to the resources required, but also because attrition rates for new cancer therapeutics are very high. Only 5% of agents that show pre-clinical promise gain a license to be used in patients after phase III trials (2). While the use of *in vitro* techniques and *in vivo* murine models are well-established in pre-clinical cancer research, fewer large animal translational models have been described. These models show promise in overcoming current limitations in pre-clinical research by permitting the integration of drug development techniques with surgical procedures and anesthetic protocols, along with novel cancer therapeutic strategies such as implantable medical devices.

The use of implantable medical devices for cancer diagnosis, treatment, and monitoring is becoming attainable due to advances in electronics and microfabrication techniques. *In vivo* murine studies have already shown that implantable devices can be used to detect cancer secreted biomarkers (3) or to release chemotherapy drugs directly within tumors (4). Numerous other studies have also investigated the biocompatibility and functionality of implantable devices using *in vivo* (predominantly rodent) models for a range of other disease conditions, providing evidence of their increasing potential for clinical uses (5).

Lung cancer remains the most commonly diagnosed cancer in the world, with ~1.8 million new cases and 1.6 million cancer-related deaths recorded each year (6). Information on the molecular basis and pathogenesis of human lung cancer continues to grow through the use of numerous *in vitro* cell line and *in vivo* murine models (7–12). However, pre-clinical research using murine models has failed to improve overall survival rates, which remains low (~15%).

Comparative oncology is the use of naturally-occurring cancers that arise in veterinary species for the study of cancer biology and therapy (13); this approach is increasingly being used to reconcile the gap between *in vitro* experiments, *in vivo* small animal research and human clinical trials. Naturally-occurring tumors within veterinary species that have incidence rates or pathological similarities comparable to human cancers have considerable potential as translational models of human disease (14). Ovine pulmonary adenocarcinoma (OPA) is a naturally-occurring neoplastic lung disease caused by the jaagsiekte sheep retrovirus (JSRV) (15–18). The disease is regarded as a valuable translational pre-clinical research model for studying human lung cancer, overcoming many of the limitations associated with current murine models (19).

The Implantable Microsystems for Personalized Anti-Cancer Therapy (IMPACT) programme (University of Edinburgh) is developing miniaturized implantable O_2_ and pH sensors designed to monitor the tissue microenvironment within a solid tumor. The identification of hypoxic tumor regions should improve the ability to target these radiation and chemo-resistant areas (20). Each sensor is fabricated on a silicon chip and bonded to a 1.7 × 200 mm long flexible printed circuit board lead. The sensors are sealed in biocompatible epoxy resin, resulting in an overall sensor size of ~2.8 × 5.1 × 1.4 mm (width × length × height). The sensors are sterilized using ethylene oxide. We have capitalized upon a naturally-occurring OPA model in order to validate these sensors within a solid tumor. By integrating techniques used in the treatment of human lung cancer patients (ultrasound, general anesthesia, CT, and surgery) into the OPA model, we have shown its translational potential. Whilst our model was specifically developed for the implantation of sensors into solid tumors, we believe it has considerable potential for other pre-clinical studies.

## Materials and Methods

Studies were undertaken under a UK Home Office Project License in accordance with the Animals (Scientific Procedures) Act 1986 and with approval from the University of Edinburgh Animal Welfare and Ethical Review Boards. The recommended guidelines for welfare and use of animals in research were followed. Nine adult female sheep (Highlander, *n* = 1; Scottish blackface, *n* = 7; Scotch Mule, *n* = 1), weighing 39–65 kg and diagnosed with naturally-occurring pre-clinical OPA, were obtained through an on-farm ultrasound eradication programme (21, 22). Sheep were bedded on straw, with *ad libitum* access to food and water in groups of at least 2 animals and were allowed a period of adaptation of at least 24 h before undergoing anesthesia.

### General Anesthesia

**Figure 1:**
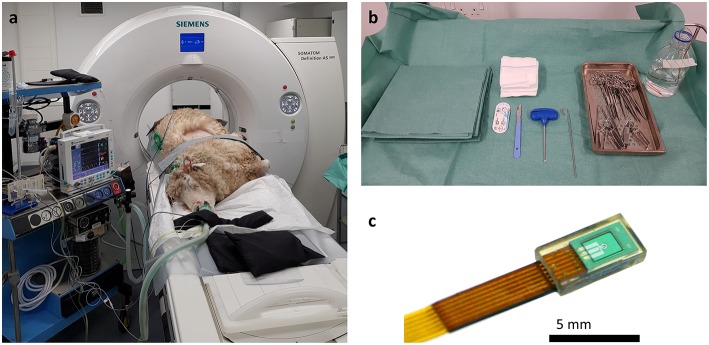
Photographs of imaging and surgical facilities. **(a)** The sheep is anesthetized and positioned for CT scanning in lateral recumbency, with the OPA affected lungs uppermost. The anesthetic monitoring equipment can be seen to the left-hand side of the image. **(b)** For sensor implantation a general surgical kit is required along with the Jamshidi insertion needle. An IMPACT sensor can be seen in a bottle of sterile saline at the top right-hand side of the image. **(c)** Macroscopic image of the IMPACT O_2_ sensor encapsulated in a biocompatible epoxy resin and bonded to the lead wire.

Anesthesia was managed by specialist veterinary anesthetists or by veterinary surgeons enrolled in a specialist training programme under supervision. All sheep underwent preanaesthetic assessment, which involved distant observation of demeanor, breathing rate and pattern, and was followed by physical examination. Only animals that were judged fit for anesthesia were subsequently studied. Food was withheld for 12 h before anesthesia, but access to water was permitted until preanaesthetic medication was administered. Anesthesia and analgesia techniques are provided in Table 1. Intravenous preanaesthetic medication was administered to reduce animal stress, facilitate the induction of anesthesia and to decrease induction agent dose requirements. General anesthesia was induced within 10 min of preanaesthetic medication to minimize sedation-induced respiratory depression. Before this, and when necessary, the head was elevated to prevent respiratory secretions and rumen contents entering the upper airway. After induction of anesthesia, the trachea was intubated with a cuffed endotracheal tube and the cuff inflated. Anesthesia was maintained using isoflurane (Abbot Animal Health, Maidenhead, UK) vaporized in an O_2_/air mixture, administered using a Bain or circle breathing system connected to the endotracheal tube. End-tidal concentrations of 1.5–2.0% isoflurane were used to ensure unresponsiveness to subsequent procedures. Oropharyngeal and tracheobronchial suction was performed to remove respiratory secretions when required. After tracheal intubation, the lungs were ventilated mechanically to achieve tidal volumes of 8–10 ml/kg. Respiratory rate was adjusted to maintain normocapnia (PaCO_2_ range 4.7–6 kPa). Body temperature was monitored using rectal and esophageal thermistors and maintained between 38.5°C and 39.5°C. A central (jugular) venous 14G cannula was used for administering drugs and crystalloid fluids. Compound sodium lactate (Aqupharm No 11, Animalcare, York, UK) was infused at 5/ml/kg/h in order to sustain cardiac preload and replace lost fluids and electrolytes. Mean arterial blood pressure was maintained between 70 and 80 mmHg and monitored using an arterial cannula placed in the central auricular artery. Blood samples obtained from the arterial cannula was used for intermittent blood-gas, biochemical and hematological analysis (Epoc portable blood gas electrolyte and critical care analyser; Woodley Equipment Company Ltd, Lancashire, UK). A multiparameter patient monitoring device (Datex-Ohmeda S/5, SOMA Technology, Madison, USA) was used to continuously monitor pulse rate and blood pressure along with pulse oximetry, capnography, temperature, spirometry, electrocardiography and inspired and expired gases (O_2_, CO_2_, and inhalant anesthetic agent) (Figure 1). Analgesic agents were administered pre-emptively either at the time of sedation or immediately post-induction. All animals were euthanized with intravenous sodium pentobarbitone (Pentoject; Animalcare, York, UK).

**Table 1:** Techniques used to provide anesthesia and analgesia in sheep with ovine pulmonary adenocarcinoma in pre-clinical research.

**Phase**	**Drug**	**Manufacturer**	**Dose (mg/kg)**	**Route**
Sedation	Medetomidine *in combination with*	“Sedator”; Dechra Veterinary Products, Shrewsbury, UK	0.003–0.01	i.v.
	Midazolam	“Hypnovel”; Roche, Welwyn Garden City, UK	0.25–0.5	i.v.
Induction	Propofol	“Propofol”; Fresenius Kabi, Cheshire, UK	To effect^*^ (e.g., 3–10)	i.v.
Maintenance	Isoflurane	“IsoFlo”; Abbot Animal Health, Maidenhead, UK		inhaled
Analgesia	Flunixin	“Flunixin Injection”; Norbrook, Newry, UK	2.2	i.v.
	Morphine	“Morphine Sulfate”; Martindale Pharmaceuticals, Essex, UK	0.1–0.3	i.v./i.m.

* *Until conditions for endotracheal intubation are present. (i.m., intramuscular; i.v., intravenous)*.

### Computed Tomography Imaging

A single-section SOMATOM Definition AS 64 slice helical CT machine (Siemens Healthcare Ltd, Camberley, UK) was used for all advanced imaging procedures. The imaging parameters of the scanner were 120 kVp, 35 mA, 3–5 mm collimation with 1 mm section thickness. The window width and level were ~2,000 and −500 HU, respectively, allowing simultaneous visualization of the needle tip, blood vessels, OPA lesions, pneumothorax, bone, muscle, and fat. All scans were performed to include the entire thoracic cavity from the thoracic inlet to the last rib.

### Development of a Trans-Thoracic Percutaneous Technique for Sensor Implantation Into OPA Tumors

The model was initially developed using cadavers of OPA-affected sheep; simulated surgeries were performed on 8 sheep cadavers with multiple sensor implantations in each carcass. These surgeries allowed the development of the implantation procedure and investigation of the potential accessible regions of the thoracic cavity and lung lobes into which sensors could be safely implanted. To refine the surgical procedure further, 3 sheep diagnosed with pre-clinical OPA by ultrasound screening underwent anesthesia and sensor implantation as developed from the cadaveric studies. Refinements to the procedure included the use of radiopaque grid lines for improved accuracy of lesion localization and performing serial CT scans to aid needle positioning and sensor implantation. These staged series of experiments allowed the development of our OPA model; each development stage increased the complexity of the model, resulting in the refined protocol used in experimental cases.

**Figure 2:**
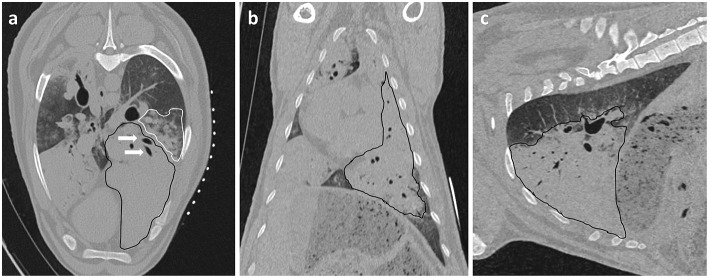
Initial CT images are used to assess intra-thoracic pathology. **(a)** Axial, **(b)** Coronal, and **(c)** Sagittal planes. A large area of increased radiopacity, consistent with an OPA lesion, can be seen affecting the ventral regions of the left cranial and caudal lung lobes (outlined in black). Air bronchograms are visible within this region (white arrows). An area of patchy and hazy increased opacity (ground glass appearance) within the dorsal regions of the lung, with preservation of bronchial and vascular patterns, can also be identified (outlined in white); this increased opacity may due to the presence of diffuse areas of neoplastic foci or pneumonia. The radiopaque circles on the skin surface seen on the axial plane are the grid lines used for OPA lesion localization.

**Figure 3:**
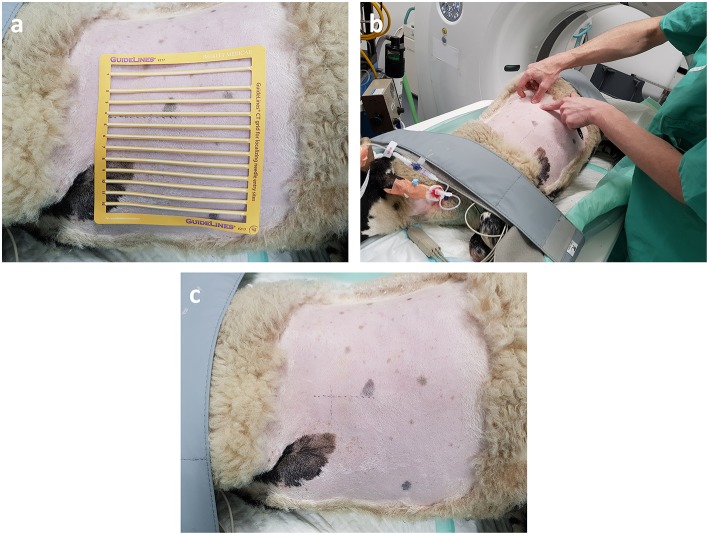
OPA lesion localization. The hemithorax has been clipped for surgery and the radiopaque grid lines are placed on the skin surface. **(a)** The grid lines are placed on the skin surface prior to the initial CT scan. **(b,c)** The skin is marked both dorso-ventrally and cranio-caudally at the desired implantation point based on the initial CT images.

All experiments were conducted on anesthetized animals. After induction of anesthesia, sheep were placed in lateral recumbency with the diseased lung uppermost. The thorax was clipped between the caudal border of the last rib and the caudal border of the scapula. The dorsal margin extended from the dorsal spinous process of the thoracic vertebrae ventrally to the sternum. An initial CT scan was taken to assess intra-thoracic pathology and identify OPA lesions for implantation. Lesions were selected so the needle path would avoid bullae, fissures, visible blood vessels, and large bronchioles. Peripheral lung lobe lesions of at least 4 cm diameter were preferred to limit the volume of normal aerated lung that the needle would pass through and to improve sensor implantation into OPA tissue (Figure 2). To aid OPA lesion localization and determine the site for percutaneous sensor placement, initial CT scans were performed with a self-adhesive sheet of non-metallic, radiopaque grid lines (GuideLines, Oncology Imaging Systems, UK) placed on the thoracic wall skin surface. OPA lesions were localized dorso-ventrally based on the grid lines and cranio-caudally based on intercostal spaces. The distance between the skin and pleura was measured at the anticipated penetration site. A mark was drawn on the skin surface to identify the position of thoracic wall penetration for sensor implantation. The grid lines were removed, and the skin was aseptically prepared for surgery using chlorohexidine solution, after which the area was four quarter draped for surgery (Figure 3).

**Figure 4:**
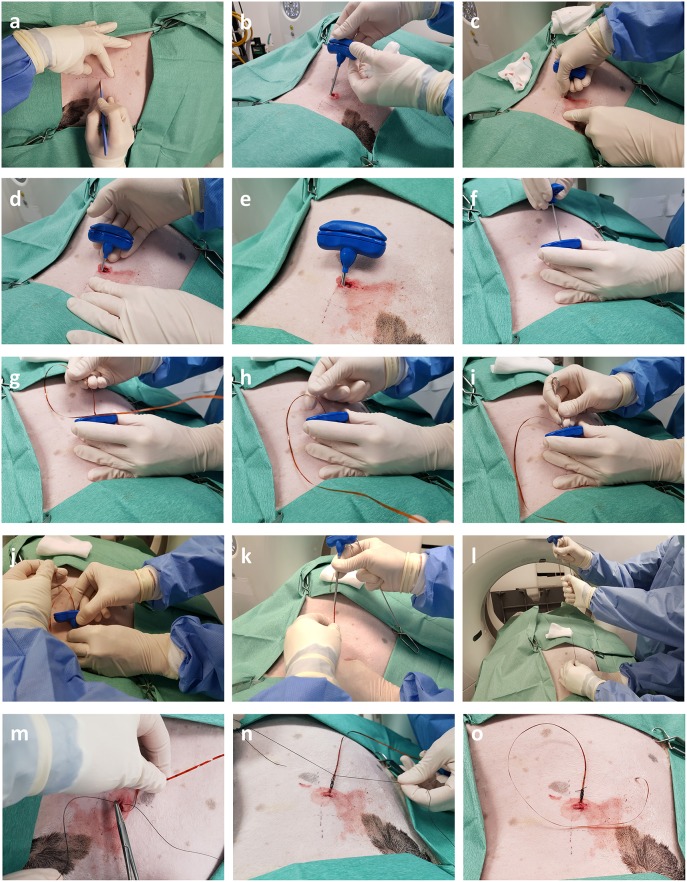
Intra-operative photographs depicting trans-thoracic percutaneous sensor placement. **(a,b)** A skin incision is made through which the Jamshidi needle is introduced. **(c–e)** Following successive CT scans the needle is progressively advanced into OPA tissue. **(f–h)** Once the needle is in position the stylet is removed and the sensor introduced down the bore of the needle. **(i)** The obturator is used to push the sensor past the tip of the needle into OPA tissue. **(j–l)** The Jamshidi needle is removed, leaving the sensor and lead wire in place. **(m–o)** The skin is closed, and lead wire secured in place with a purse string and Chinese finger trap suture.

**Figure 5:**
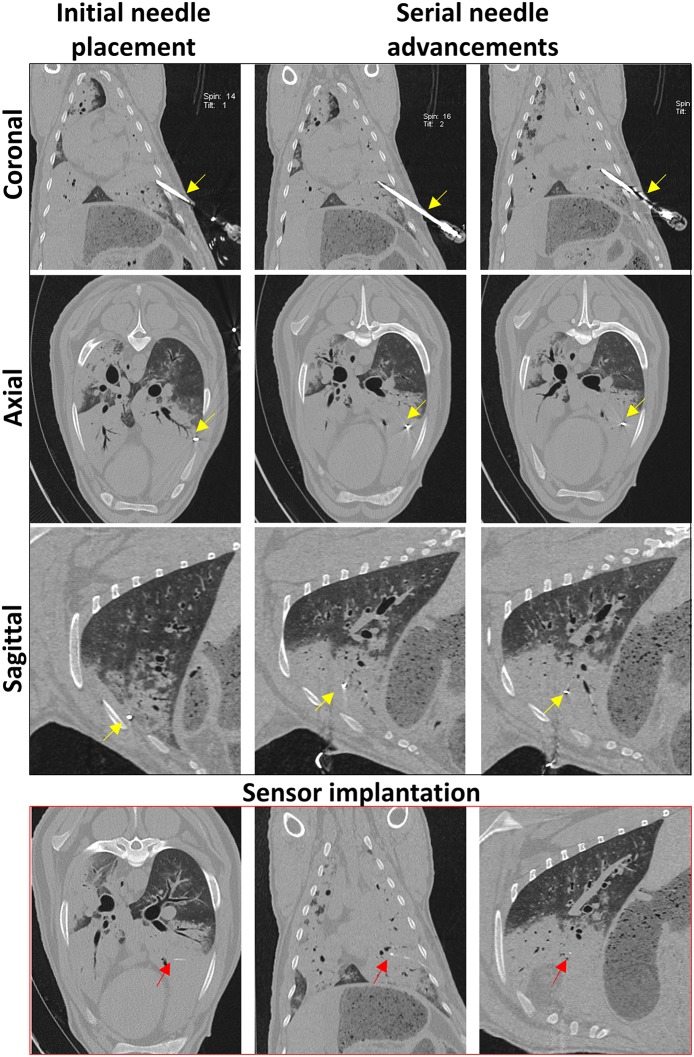
Serial CT images taken during sensor implantation. Coronal, axial, and sagittal planes are viewed following initial needle placement and after each needle advancement. The Jamshidi needle (yellow arrows) is advanced until the tip is positioned at the desired point within the OPA lesion. CT images taken immediately post-implantation demonstrates sensor placement within OPA tissue in all 3 planes (red arrows).

All sensors were inserted using a trans-thoracic percutaneous technique under CT guidance. Based on the initial pre-operative CT scan a 1 cm vertical skin incision was made ~1–2 intercostal spaces caudal to the desired entry point into the thoracic cavity. An 8G ×15 cm Jamshidi biopsy needle (Carefusion, France), with its stylet in place, was advanced cranially through subcutaneous tissues, then redirected perpendicular to the thoracic wall in the center of the chosen intercostal space. The needle was advanced through the chest wall (based on the pre-measured distance from the initial CT scan), with the penetration of the parietal pleura appreciated as the feeling of a “pop.” The needle, at this point, was within the thoracic cavity through the parietal pleura, but not penetrating lung/OPA tissue. A second CT scan at this stage confirmed the position of the needle. If necessary, the needle could be repositioned with minimal risk of lung damage as the needle had not penetrated the visceral pleura. Once in the correct position the needle was slowly advanced through the visceral pleura into OPA tissue; repeat CT scans were taken following each needle advancement and measurements were made determining the distance from needle tip to the point of desired sensor implantation. Following placement of the needle tip centrally within OPA tissue, the stylet was removed from the Jamshidi needle and the sensor and lead wire were introduced down the bore of the needle. The obturator was then placed down the bore of the needle, advancing the sensor past the tip of the needle into OPA tissue. Once in place, the obturator and implantation needle were withdrawn, leaving the sensor and lead wire *in situ*. A purse string suture of 3 metric braided silk (Mersilk, Ethicon UK), placed around the incision which continued as a Chinese finger trap suture around the lead wire, secured the sensor in place (Figures 4, 5). Final CT scans were performed to evaluate sensor positioning and assess any immediate post-operative complications such as pneumothorax or hemorrhage. The decision to drain any pneumothorax that developed (though percutaneous thoracocentesis) was made based on its severity. Post-mortem examination was performed following the completion of the experiments to assess the extent of lung pathology, identify the implant site and to obtain biopsy specimens for histopathology.

### Histopathology

OPA tissue was fixed for at least 24 h (depending on tissue thickness) in 4% formaldehyde (Genta Medical, UK) before undergoing processing using the Thermo Scientific Excelsior AS Tissue Processor (Thermo Scientific, UK) and embedding in paraffin. Tissue was sectioned using the Leica RM2235 rotary microtome (Leica Microsystems Ltd, UK); microtome sections of 4 μm were placed on SuperFrost Plus glass slides (Thermo Scientific, UK) and allowed to dry for a minimum of 4 h at 53°C.

For haematoxylin and eosin staining, sections were deparaffinised by 3 changes in 100% xylene for 5 min, then rehydrated by placing into alcohol; 2 changes in 100% ethanol, followed by 80% then 50% for 2 min each time. The slides were washed in running water for 2 min, before placing in haematoxylin (Shandon Harris Haematoxylin, Thermo Scientific, UK) for a maximum of 10 min. Slides were washed in running water for 2 min and then placed into Scott's tap water substitute for a maximum of 10 min until the tissue sections turned blue. Sections were counterstained by placing them into Eosin Y (Shandon Eosin Y Cytoplasmic Counterstain, Thermo Scientific, UK) for 5 min. The slides were dehydrated by placing them into alcohol; 50% ethanol for 30 s, 80% ethanol for 30 s, then 2 changes in 100% ethanol for 2 min. The slides were placed in xylene for 10 min before being mounted with coverslips using DXP mountant (Sigma-Aldrich, UK).

### Assessment of Radiation Exposure During CT-Guided Sensor Implantations

To assess the amount of radiation that sheep were exposed to during CT-guided sensor implantations, the total number of CT imaging events (topograms or full thoracic scans) were recorded and individual imaging event and total dose length products (DLP) were calculated for each sheep. Individual event DLP is calculated from the CT dose index volume (CTDI_vol_), which is in turn based on the radiation received inside a phantom from a single rotation of the scanner, this value is then multiplied by the scan length. Total DLP for each sheep was calculated from the sum of all individual DLP's. DLP is proportional to the effective dose received by a patient and is used, in combination with CTDI, to compare scanning protocols and establish diagnostic reference levels (23).

### Statistical Analysis

Data for blood-gas, biochemical and hematological analysis was analyzed with parametric tests. One-way ANOVA with Holm-Šídák multiple comparisons test were used to test for differences over time; *p*-values < 0.05 were deemed statistically significant. Data are shown as mean ± SEM, with all statistical analysis and graphs generated using Prism 7 (GraphPad Software, San Diego, CA, USA).

## Results

### Appropriate Anesthetic Protocols Enable OPA Sheep to Remain Physiologically Stable Throughout Anesthesia

**Figure 6:**
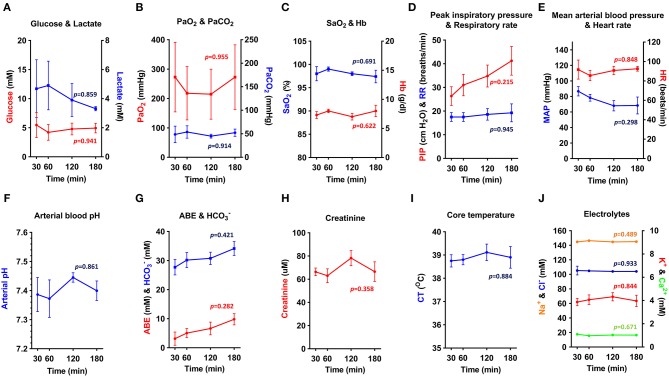
**(A–J)** Physiological parameters and arterial blood biochemical and hematological analysis. Physiological data obtained from OPA cases maintained at FiO_2_ of 1.0 throughout anesthesia (one-way ANOVA with Holm-Šídák multiple comparisons test; data expressed as mean ± SEM, *n* = 3–5 per time point; times indicate min post-induction of anesthesia) (PaO_2_, arterial O_2_ partial pressure; PaCO_2_, arterial CO_2_ partial pressure; SaO_2_, hemoglobin O_2_ saturation; Hb, hemoglobin; PIP, peak inspiratory pressure; RR, respiratory rate; MAP, Mean arterial blood pressure; HR, heart rate; ABE, arterial base excess; HC${\text{O}}_{3}^{-}$, bicarbonate; CT, core temperature).

To assess the physiological stability of OPA-affected sheep throughout anesthesia, data from blood-gas, biochemical, and hematological analysis was combined with variables such as heart rate, respiratory rate, body temperature, and mean arterial blood pressure. Results from sheep maintained with an inspired fraction of O_2_ (FiO_2_) of 1.0 are shown in Figure 6 (*n* = 3–5 per time point). The remaining 4 cases in this study were subjected to alterations in FiO_2_ for sensor validation experiments and are therefore not included in this analysis. Results showed that physiological and arterial blood variables remained stable throughout anesthesia. No statistically significant changes over time were identified in any measured variable (Figure 6).

Elevated blood lactate persisted throughout anesthesia but showed a tendency to reduce at later time points (Figure 6A). Blood pH, base excess and bicarbonate showed a similar, but opposite response (Figures 6F,G). Arterial O_2_ partial pressure (PaO_2_) showed marked individual variation (Figure 6B) and was consistently lower than expected given the FIO_2_ of 1.0, suggesting a compromise in the degree of O_2_ uptake by the alveoli. Despite this, it was possible to maintain a hemoglobin O_2_ saturation (SaO_2_) of ≥ 95% (Figure 6C). Peak inspiratory pressure increased throughout anesthesia (Figure 6D), with mean peak inspiratory pressures at 180 min almost 1.5 times greater than that recorded at 30 min. Airway suction was frequently required to clear respiratory secretions. To support mean arterial blood pressure (Figure 6E), 3 sheep required management with intravenous fluids or vasopressors. Additional treatments administered during anesthesia included atropine (1 sheep; severe bradycardia), sodium bicarbonate (1 sheep; acidosis), and glucose (1 sheep; hypoglycaemia).

### CT-Guided Trans-thoracic Percutaneous Sensor Implantation Resulted in a High Success Rate of Delivery of Sensors Into OPA Lesions

**Figure 7:**
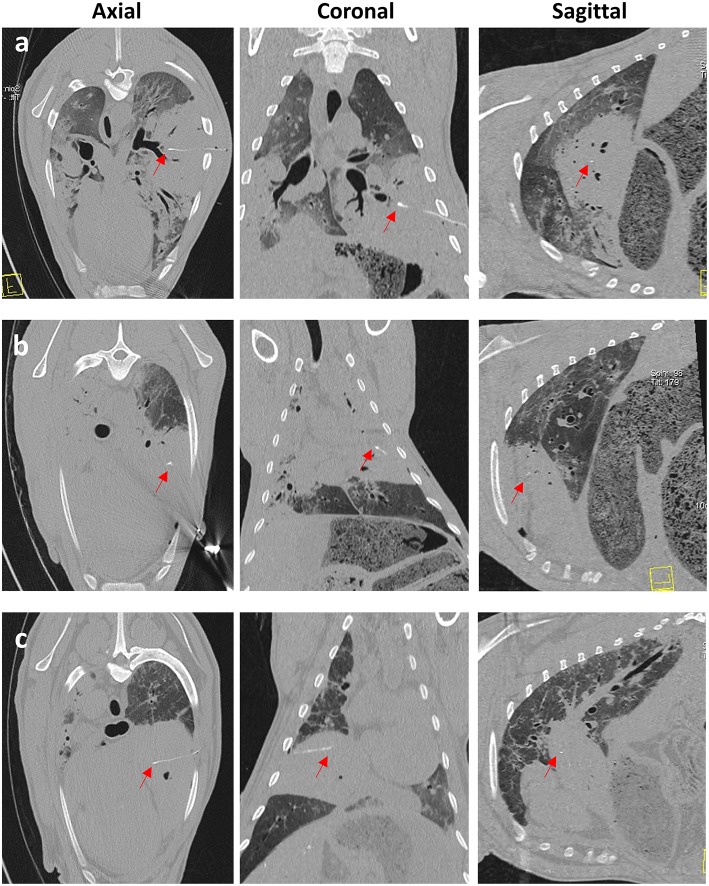
CT images taken immediately post-sensor implantation. Images **(a–c)** are from 3 separate OPA cases. The sensor and lead wire (red arrows) can be seen within the area of increased radiopacity, consistent with a large OPA tumor.

A total of 9 sheep underwent general anesthesia and sensor implantation (2 additional cases were excluded from analysis due to a lack of histological evidence of OPA following post-mortem examination). Of the 9 OPA-affected sheep that underwent CT-guided sensor implantations into tumor tissue, 7 cases received a single sensor implantation and 2 cases received 2 sensors implanted into a single large OPA lesion. In the case of single sensor implantations, time taken from the initial CT scan to sensor placement was 45 ± 5 min (mean ± SEM). Double implantations took a little longer, with implant times of 50 and 73 min for each case. The number of sequential CT scans and needle advancements required from the initial needle placement to obtaining the desired position within OPA tissue ranged from 3 to 5, with 4 advancements required in 9 of the 11 sensor implantations. All implantation procedures resulted in sensor placement within OPA tissue (Figure 7). No immediate complications were identified in 5 of the cases (Table 2). Estimates of the amount of radiation received by each sheep undergoing CT-guided sensor implantation were calculated. DLPs for individual topograms and full thoracic scans were 9 ± 0.3 mGy cm and 392 ± 11 mGy cm, respectively (mean ± SEM), whereas total DLP for each sheep was 2,856 ± 392 mGy cm (mean ± SEM).

**Table 2:** Details of OPA cases used and implantation results.

**Case**	**Signalment**	**Weight (kg)**	**CT lesion localization**	**No. of needle advancements**	**Time required for implantation (min)**	**Complications post-sensor placement**
1	Highlander Female Adult	65	Left caudal lobe: 1 focal lesion at cranial aspect of caudal lobe, ~4–5 cm diameter	4	31	Mild pneumothorax: not treated
2	Blackface Female Adult	51	Right caudal lobe: 1 diffuse area at caudal aspect ~4–5 cm diameter	3	58	Mild pneumothorax: treated successfully with a single thoracocentesis
3	Blackface Female Adult	39	Left caudal lobe: Entire lobe affected	4	46	None
4	Blackface Female Adult	52	Left cranial lobe: Entire lobe affected	4	56	None
5	Blackface Female Adult	57	Right cranial lobe: Almost entire lobe affected	4	26	None
			Left caudal lobe: 1 Focal lesion at caudal aspect of caudal lobe, ~10–15 cm diameter			
6	Blackface Female Adult	39	Left cranial lobe: Entire lobe affected	4	60	Mild pneumothorax: not treated
7	Blackface Female Adult	55	Right cranial lobe: Entire lobe affected	5	43	None
			Left cranial lobe: Entire lobe affected, extending into the cranial aspect of the left caudal lobe			
8	Blackface Female Adult	58	Right accessory lobe: Entire lobe affected	4	73	None
			Left cranial lobe: Entire lobe affected, extending into the cranial aspect of the left caudal lobe			
9	Mule Female Adult	64	Right cranial lobe: Almost entire lobe affected	4	50	Mild pneumothorax: treated successfully with a single thoracocentesis

*Cases 1–7 had single sensor implantations, whereas cases 8 and 9 had 2 sensors implanted. Signalment, CT localization, time from initial CT scan to sensor implantation, number of CT scans/needle advancements required, and immediate post-implantation complications are provided*.

### Iatrogenic Pneumothorax Is a Potential Complication Following Percutaneous Sensor Implantation

**Figure 8:**
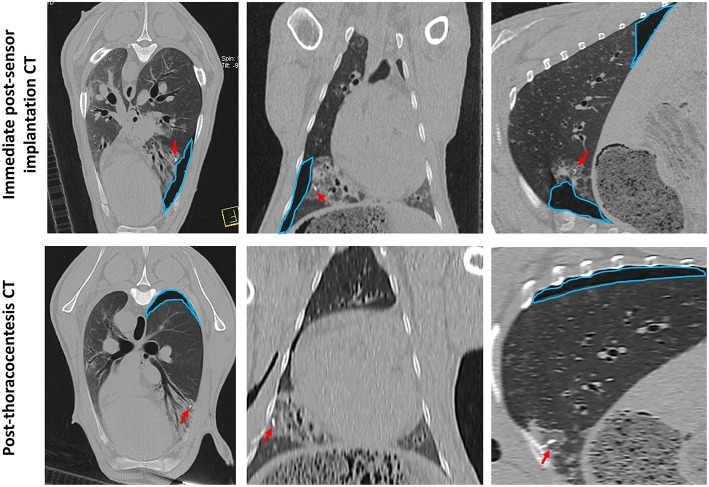
CT images documenting iatrogenic pneumothorax. The OPA target lesion is seen as a patchy area of increased radiopacity affecting the ventral region of the left caudal lung lobe. CT scans were taken immediately post-sensor implantation and following thoracocentesis. A mild pneumothorax (outlined in blue) was evident immediately post-sensor implantation, predominately localized to the region of the implantation site. The sensor can be seen within OPA tissue (red arrows). Thoracocentesis was performed immediately post-sensor placement, which resulted in lung lobe re-expansion and removal of most of the air from within the thoracic cavity; however, a small pneumothorax at the dorsal aspect of the thoracic cavity was still evident (outlined in blue).

Sensor implantation in 4 cases immediately resulted in mild pneumothoraces; however, only 2 of these cases required treatment with percutaneous thoracocentesis. CT scans post-thoracocentesis confirmed lung lobe re-expansion and removal of most of the air from within the thoracic cavity. Sensor positioning was not affected by the occurrence of a pneumothorax and the sensor remained within the OPA lesion post-thoracocentesis (Figure 8).

### All Implantation Sites Were Identified During Post-mortem Examination

**Figure 9:**
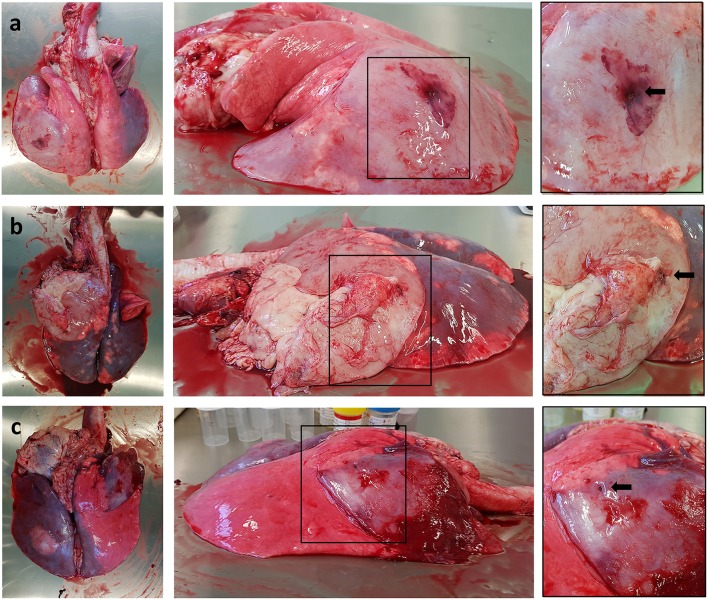
Photographs depicting gross pathology seen in typical OPA cases. Each implantation site is highlighted with a black arrow. **(a)** Large gray consolidated mass affecting the majority of the left caudal lung lobe. An area of petechial hemorrhage can be seen on the surface of the lung surrounding the needle entry point. **(b)** Large gray consolidated mass affecting the majority of the left cranial lung lobe. Fibrous tissue can be seen adherent to the lung surface just cranial and ventral to the needle entry point. **(c)** One large dark colored mass affecting the right cranial lung lobe containing the implant site, with a further focal lesion within the left caudal lung lobe.

**Figure 10:**
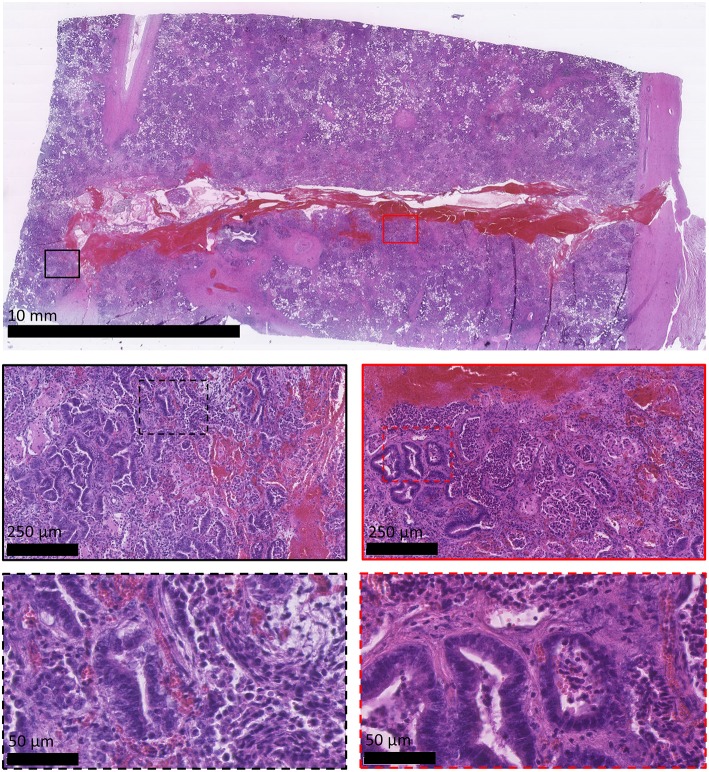
Histological appearance of OPA sensor implantation site. All images are haematoxylin and eosin stained sections. OPA tissue including the implantation site was obtained during the post-mortem examination. The top image shows the needle tract penetrating through the visceral pleura (right-hand side of image) and into the OPA tissue. Large numbers of erythrocytes can be seen within the needle tract. Higher magnification images document erythrocytes extending up to 250 μm from the implant site, predominately within stromal tissue.

All sheep underwent post-mortem examination following euthanasia. Gross pathology allowed assessment of lung pathology, identification of the implant site, and provided the opportunity to obtain biopsy specimens for histological analysis. Gross pathology identified lesions that were in accordance with those identified on the CT scans in terms of number of lesions, location, and size. All sensor implantation sites were successfully identified with an entry site seen in the visceral pleura directly overlying OPA tissue. In 1 case an area of petechial hemorrhage was evident to the lung surface in the region of the implantation site; however, the remaining cases had no gross evidence of parenchymal hemorrhage or haemothorax (Figure 9). Following examination of gross pathology, the implant site was dissected from the OPA tissue. The biopsy specimen was used for both OPA diagnosis and to assess the effects of the implantation procedure on OPA/lung tissue. Histological examination confirmed OPA diagnosis in all 9 cases (the 2 excluded cases were reported as lung consolidation with marked pleural fibrosis and pleuritis). Evidence of hemorrhage within the needle tract and erythrocytes present within tumor tissue immediately adjacent to the implant site were identified in all cases (Figure 10).

## Discussion

Similarities between OPA and human lung adenocarcinomas in terms of disease presentation, progression, and histological classification has led to the recognition of OPA as an excellent model for studying human lung cancer biology (24). *In vitro* (25–29) and *in vivo* (30–35) OPA experimental models are well-documented and have been successfully used to identify molecular pathways involved in lung cancer pathogenesis. However, for OPA to be used as a translational pre-clinical research model for human lung cancer, techniques used in the diagnosis and treatment of human patients must be incorporated into the model. In order to achieve this aim, protocols currently used in human thoracic medicine were incorporated into our novel OPA model, which was used for validation of the sensors which have been developed as part of this project (20).

Although certain thoracic procedures in human medicine are commonly performed under local anesthesia, general anesthesia was mandatory in our OPA model to ensure animal and personnel safety. It is therefore important to consider the general anesthetic requirements of these sheep if they are to be used in translational research. General anesthesia of sheep with OPA can be challenging but is entirely feasible if facilities and expertise are in place to provide, if required, respiratory, and cardiovascular support. These animals have variable amounts of respiratory compromise resulting from the OPA lesion(s), lung lobe consolidation, increased respiratory tract secretions, secondary infections, and anesthesia-induced atelectasis. All these factors will hinder the effective movement of inspired O_2_ into the blood, leading to lower PaO_2_ levels. Adequate blood O_2_ content in our cases was maintained by increasing the inspired fraction of O_2_ in combination with mechanical ventilation. The use of elevated peak inspiratory pressures in our cases was well-tolerated and was necessary to achieve adequate ventilation due to reduced compliance of the diseased lungs. The sheep in our study also had elevated blood lactate levels, which could have been caused by global tissue hypoxia; however, as neoplasia itself can elevate blood lactate levels (36) it is difficult to know its specific underlying etiology. The decrease in lactate levels that occurred throughout anesthesia may have been due to the provision of intravenous fluid therapy and mechanical ventilation which contribute to improved tissue O_2_ delivery. Although we have shown that OPA cases may require additional anesthetic monitoring with respiratory and cardiovascular support, all our cases were successfully managed throughout sensor validation experiments. These results provide evidence that OPA cases, even with relatively large tumors (as was seen with a number of our cases), can be used in procedures that require general anesthesia. Although, it should be noted that these were pre-clinical OPA cases identified by ultrasound screening and sheep showing clinical signs of OPA were specifically excluded from the study.

Lung cancer diagnosis in human patients is performed through immunohistochemistry using aspirates or biopsy samples taken using a flexible bronchoscope (37), or via a minimally-invasive trans-thoracic approach (38–40). The choice of which technique to use is dependent on the location of the lesion. Central lesions involving a bronchus will be readily assessible with a bronchoscope, whereas peripheral lesions that are either not visible on endobronchial examination (41), <3 cm in diameter or those that do not show a bronchus entering the lesion on CT images will be more suited to minimally invasive trans-thoracic needle biopsy (TTNB) (38, 39, 42). Both endoscopic and percutaneous biopsy techniques could have been modified for use in our OPA model; however, for several reasons the trans-thoracic percutaneous approach was chosen. Naturally-occurring JSRV infection and transformation will typically result in OPA lesions forming initially at peripheral lung lobe regions. It is only as neoplastic foci enlarge and coalesce that central lobe regions become affected. Although tumor tissue will frequently involve bronchioles, larger bronchi may remain largely unaffected. Although endoscopy can be routinely performed in sheep (43), successful endoscopic sensor implantation would only be possible in tumors which involved bronchi of sufficient diameter that could accommodate an endoscope. This specific set of selection criteria would limit the number of cases that could be used, and logistically could only be assessed once a sheep is anesthetized and CT images have been obtained. As OPA tumors can be associated with significant volumes of lung fluid production, present in the large and small airways, this would hamper endoscopic airway visualization and make sensor implantation extremely challenging. The sensor for which we developed the model is currently wired therefore endoscopic implantation would require the lead wire to run up through the large airways and out through the larynx to be connected to external instrumentation; the presence of the endotracheal tube would make this almost impossible. These limitations associated with endoscopic sensor delivery led us to develop the minimally invasive trans-thoracic percutaneous approach for sensor implantation.

In human medicine TTNB requires the use of image guidance. Fluoroscopy, once the preferred imaging choice, enables needle advancements to be visualized in real-time (44); however, the technique has become less popular as it is not compatible with accessing deep lesions and the avoidance of vascular structures and bullae (45). Image guidance using ultrasound enables needle movements to be monitored precisely and quickly during the TTNB (46, 47); however, its use is restricted to peripheral lesions that produce an acoustic window. CT is currently the most commonly used image guidance technique for TTNB (48–50). Unlike fluoroscopy, CT allows accurate planning of needle path trajectories that avoid aerated lung, bullae, fissures, and blood vessels. The procedure can also be used to sample central lesions, lesions <1 cm in diameter (51) and, similar to ultrasound, can distinguish between necrotic and solid regions of a lesion, allowing for more accurate needle positioning and better diagnostic samples to be obtained. CT can be combined with fluoroscopy (CTF) to allow needle adjustments to be made in almost real-time. The technique is primarily used for very small lesions located in difficult to access thoracic regions (costodiaphragmatic recess, near to the mediastinum or critical at-risk structures) and can be performed quickly, which is advantageous in un-cooperative or high-risk patients (52). Although any of these image techniques can be integrated for use within the OPA model, CT was chosen in our study for several reasons. CTF was not considered necessary as sheep were selected for use in our experiments on the basis that they had reasonably large OPA lesions in relatively accessible lung regions. CTF would also have required the use of lead aprons and radiation shields for safety purposes. Although ultrasound guidance could have been used for sensor implantations into OPA lesions affecting pleural surfaces, the technique could not provide an assessment of pathological lesions occurring throughout the entire thorax, and thus cannot be used to aid the selection of the most appropriate lesion for implantation. These factors directed us to use CT guidance for sensor implantations.

Serial CT scans were performed during the implantation procedure, with each CT scan reviewed at each stage of the process. The initial CT scan was used to select the OPA lesion to undergo sensor implantation, while sequential scans were used to assess needle trajectory and position. Lesion selection and needle path planning was based on known risk factors associated with the development of TTNB complications in human patients, predominantly pneumothorax and hemorrhage. Small lesions and the presence of emphysema (53) or chronic obstructive pulmonary disease (54) can increase complication rates. Although it is possible that these diseases can occur with OPA, we did not see any evidence of them based on CT imaging; therefore, their association with complications seen in the OPA model is likely to be low. Technical factors associated with performing TTNB are also known to influence the occurrence of post-operative complications, these factors are likely to have played a more significant role in the complication rate seen in our model. Technical factors that can increase the risk of TTNB complications include increased amounts of normal aerated lung crossed by the needle (55, 56), a small oblique needle angle with the thoracic pleura (57), repositioning the needle multiple times (58), a greater number of sampling procedures (59), the absence of previous ipsilateral surgery (60), using a trans-fissure approach (56) and damage to thoracic vasculature. In accordance with these known risk factors, lesions were chosen so that the needle path avoided passing through bullae, large blood vessels, bronchi and interlobar fissures. If more than one lesion was present, a peripheral lesion was chosen to decrease the amount of lung tissue that would be traversed (61). It is interesting to note that procedural length or needle dwell time within the lung is not associated with increased risk of pneumothorax (59). In our model single sensor implantations were performed in a time of 45 ± 5 min (mean ± SEM), similar to studies in human patients that document CT-guided TTNB times of up to 66 min (59).

In human medicine, monitoring and standardization of radiation dose from diagnostic and interventional procedures is now commonly performed in an effort to minimize potential risks to patients from radiation exposure (23). In our model the mean DLP for a single full thoracic scan was 392 ± 11 mGy cm (mean ± SEM), which is comparable with recommended diagnostic reference levels of 517 mGy cm used for human thoracic CT scans (62). As previously described, multiple CT scans were performed during sensor implantations, which resulted in a total mean DLP of 2,865 ± 392 mGy cm (mean ± SEM). This value is higher than that reported in the human literature for patients undergoing CT-guided TTNB, with one study documenting a total mean DLP of 801 mGy cm (63). However, in the same study DLPs as high as 3,684 mGy cm were reported for patients undergoing thoracic drainage procedures. The relatively high total DLPs observed in our study was due to the need to obtain high quality images for accurate needle path planning and sensor placement, which in combination with additional post-sensor implantation scans, will have increased the total radiation dose received by each sheep. However, as our results have shown that single thoracic scans were lower than human recommended diagnostic reference levels and reports for patients undergoing thoracic drainage, this provides evidence that CT-guided techniques in sheep are comparable with similar human procedures.

Following the implantation procedure, a CT scan was performed to assess for complications and to evaluate final sensor positioning. Although numerous TTNB associated complications have been documented which include infection, air embolism, lung lobe torsion, and needle tract metastasis, by far the most common complications are pneumothorax and hemorrhage (64).

Although pneumothorax rates as high as 54% have been documented (65, 66), accepted occurrence rates are more likely to be in the region 17–26%, of which ~14% will require percutaneous aspiration or chest tube insertion (57, 60, 67, 68). In our series of experiments, 4 out of 9 cases (44%) developed a mild immediate post-implantation pneumothorax, however only 2 required percutaneous needle thoracocentesis. The cases that received thoracocentesis resulted in lung lobe expansion, with the sensor remaining within the OPA lesion. In each case the pneumothorax did not reoccur; this was likely due to the removal of excess pleural air from around the implant site, allowing apposition of visceral and parietal pleural surfaces. Measures that were used to reduce the risk of pneumothorax included the use of a coaxial needle, to allow the sensor to be placed with a single pleural puncture, and careful needle path planning.

Although hemorrhage is the second most common TTNB complication that occurs in ~4–10% of cases (67, 69), the development of a haemothorax is <1% (64). Bleeding may be identified through blood coming up through the bore of the needle, or as a ground-glass appearance on CT images typically in the region of the biopsy or along the needle path. Measures that were used to reduce the risk of hemorrhage included the avoidance of large pulmonary and cardiovascular vessels. Placing the needle through the center of the intercostal space also reduced the risk of damage to intercostal neurovascular bundles. In our study no cases were identified as having post-implantation hemorrhage based on CT image evaluation; however, following post-mortem examination and implant site histopathology erythrocytes were identified within the needle tract in all cases. This finding is not unexpected as tumor tissue can have an extensive blood supply. Passage of a large needle through tumor parenchyma will inevitably damage intra-tumoural macro and microvessels. This situation is also likely to occur in human patients undergoing TTNB, but as the needle tract itself would never be biopsied there is no available data to support this. Limited amounts of erythrocytes were identified in OPA tissue away from the needle tract itself. It is possible that the OPA tissue immediately adjacent to the implant site and needle tract has reduced compliance compared with normal lung tissue, and could potentially act as a tamponade, preventing the escape of erythrocytes into the tumor tissue. The under reporting of alveolar hemorrhage based on the CT images is likely due to the appearance of the OPA lesions themselves. All cases had large OPA lesions involving the visceral pleura that were characterized as having increased radiopacity, frequently with the presence of air bronchograms. This CT appearance of typical OPA tumors would have likely obscured any hemorrhage that occurred within the needle tract itself.

The large needle size required for sensor implantation probably contributed to the complication rate encountered in the model. The needle diameter had to exceed that of the IMPACT sensor and lead wire. Consequently, an 8G Jamshidi needle was selected, which is considerably larger than the 18–22G needles that would be routinely used in human TTNB procedures. Ongoing development and miniaturization of the sensors will allow smaller diameter needles to be used. Although smaller diameter needles may reduce the occurrence of complications, our current model has shown a comparable complication rate to that seen in human patients undergoing TTNB.

Although not considered within this article, the primary aim of developing this novel OPA model was to validate the *in vivo* functionality of the IMPACT O_2_ and pH sensors within a tumor microenvironment. In order to achieve this goal, we performed a series of physiological challenges following sensor implantation. These challenges included altering blood oxygenation levels through FiO_2_ manipulations and varying blood pH levels though administering inhaled CO_2_ and altering ventilation rates. The results of sensor validation experiments will be published in separate articles and will highlight the ability of the OPA model to be used to validate novel medical technologies.

## Conclusion

This paper has described the use of naturally-occurring pre-clinical OPA cases in the development of a novel *in vivo* ovine model for the CT-guided trans-thoracic percutaneous implantation of sensors into OPA tumors. This is, to the best of our knowledge, the first description of the use of naturally-occurring OPA cases as a surgical model. Through the integration of techniques such as ultrasound, general anesthesia, CT and surgery into the OPA model, we have demonstrated its translational potential and effectiveness as a pre-clinical research tool for human lung cancer. We have also shown our model to be comparable to TTNB in human patients in terms of procedure duration, radiation exposure, and complication rate. We believe this model can be developed further for other pre-clinical uses, such as the procurement of biopsy specimens, the development of medical devices for the local delivery of chemotherapeutic agents, monitoring the tumor microenvironment and in the assessment of the effectiveness of RT or systemic chemotherapeutic agents. This model has great potential to not only advance the molecular understanding of human lung cancer, but to also improve pre-clinical research and enhance the treatment of human lung cancer patients.

## Data Availability

All datasets generated for this study are included in the manuscript.

## Ethics Statement

Studies were undertaken under a UK Home Office Project License in accordance with the Animals (Scientific Procedures) Act 1986 and with approval from the University of Edinburgh Animal Welfare and Ethical Review Boards. The recommended guidelines for welfare and use of animals in research were followed.

## Author Contributions

DA and AM secured funding for this research and conceptualized the initial work. MG with contributions from PS developed the surgical procedure. SG, RC, and RG conducted all anesthetic procedures. JRKM performed all engineering work. MG wrote the majority of the manuscript and composed the figures, with contributions from JM and SG who wrote parts of the introduction and anesthesia sections respectively. CC and DG were involved with obtaining pre-clinical OPA cases. Critical revisions were made by all authors. All authors read and approved the final manuscript.

### Conflict of Interest Statement

The authors declare that the research was conducted in the absence of any commercial or financial relationships that could be viewed as a potential conflict of interest.
